# General Health Statuses as Indicators of Digital Inequality and the Moderating Effects of Age and Education: Cross-sectional Study

**DOI:** 10.2196/37845

**Published:** 2022-10-21

**Authors:** Alexander J A M van Deursen

**Affiliations:** 1 Department of Communication Science University of Twente Enschede Netherlands

**Keywords:** digital inequality, health, MOS, eHealth, digital health, online health, age, education, survey, digital divide, attitude, health outcome, patient outcome, internet access, internet skill, technology skill

## Abstract

**Background:**

Considerable effort has been directed to offering online health information and services aimed at the general population. Such efforts potentially support people to obtain improved health outcomes. However, when health information and services are moved online, issues of equality need to be considered. In this study, we focus on the general population and take as a point of departure how health statuses (physical functioning, social functioning, mental health, perceived health, and physical pain) are linked to internet access (spanning internet attitude, material access, internet skills, and health-related internet use).

**Objective:**

This study aims to reveal to what extent (1) internet access is important for online health outcomes, (2) different health statuses are important for obtaining internet access and outcomes, and (3) age and education moderate the contribution of health statuses to internet access.

**Methods:**

A sequence of 2 online surveys drawing upon a sample collected in the Netherlands was used, and a data set with 1730 respondents over the age of 18 years was obtained.

**Results:**

Internet attitude contributes positively to material access, internet skills, and health outcomes and negatively to health-related internet use. Material access contributes positively to internet skills and health-related internet use and outcomes. Internet skills contribute positively to health-related internet use and outcomes. Physical functioning contributes positively to internet attitude, material access, and internet skills but negatively to internet health use. Social functioning contributes negatively to internet attitude and positively to internet skills and internet health use. Mental health contributes positively to internet attitude and negatively to material access and internet health use. Perceived health positively contributes to material access, internet skills, and internet health use. Physical pain contributes positively to internet attitude and material access and indirectly to internet skills and internet health use. Finally, most contributions are moderated by age (<65 and ≥65 years) and education (low and high).

**Conclusions:**

To make online health care attainable for the general population, interventions should focus simultaneously on internet attitude, material access, internet skills, and internet health use. However, issues of equality need to be considered. In this respect, digital inequality research benefits from considering health as a predictor of all 4 access stages. Furthermore, studies should go beyond single self-reported measures of health. Physical functioning, social functioning, mental health, perceived health, and physical pain all show unique contributions to the different internet access stages. Further complicating this issue, online health-related interventions for people with different health statuses should also consider age and the educational level of attainment.

## Introduction

### Background

The World Health Organization (WHO) stresses that public health is an important topic on policy agendas in most Western countries. Considerable effort is directed to offering health information and services aimed at the general population online. Such efforts potentially support people in improved outcomes regarding their knowledge of health issues, health communication with professionals, decision-making about health issues, proper use of health services, and improved ways of taking care of themselves [1-3]. However, when health information and services are moved online, issues of equality need to be considered. Online information and services can also disempower marginalized people by violating their rights and autonomy [4], further entrenching their position. Digital inequality research typically considers how specific populations can benefit from access to online services and has shown that those most likely to experience health-related issues are also less likely to benefit from the internet in general [5]. In this respect, most attention has focused on, for example, age, racial and ethnic, and socioeconomic differences in access to online health. Actual well-being in terms of personal health is far less studied as a determinant of internet access in digital inequality research [5]. When considered, it is often simplified in binary terms or by a single self-rated health scale. In this study, we focus on the general population and take as a point of departure the way people with different health statuses—pertaining to general functioning and well-being—use the internet to obtain positive health outcomes, for example, in determining a medical condition from which one might suffer or making better health-related decisions. We attempt to provide an in-depth picture by focusing on different health statuses in relation to stages of internet access and online health outcomes. The paper is structured around 3 goals: to reveal to what extent (1) internet access (spanning internet attitude, material access, internet skills, and internet health use) is important for online health outcomes, (2) different health statuses (physical functioning, social functioning, mental health, perceived health, and physical pain) are important for obtaining internet access and outcomes, and (3) age and educational differences moderate the contribution of health statuses to internet access.

### Internet Access and Outcomes

Resources and appropriation theory considers internet access as a process of appropriation following attitude, material access, skills, and use [6]. A positive attitude toward the internet is a first step toward using online health information and services [6]. Subsequently, material access involves having an internet connection and the required devices that provide internet access, such as desktops, laptops, tablets, and smartphones [6,7]. With the rapid increase in internet connections in Western countries, differences in materials (variety and quality of devices) are increasingly the topic of attention in this stage [7]. The required skills to use the internet range from operational skills (basic operations to use the internet) to information navigation (find, select, and evaluate sources of online information), communication (use online communication and interactions to understand and exchange meaning and acquire social capital), and content creation skills (create different types of quality content) [8]. The final access type in the current context involves the use of different types of online health apps available to the general population.

Prior research has revealed that internet attitude directly affects material access, the development of internet skills, and internet use [9]. Material access has significant relationships with both internet skills and internet use. Individuals with desktop computers, laptops, tablets, smartphones, and smart devices connect to the internet everywhere and at all times of the day and get more opportunities to develop varied skills and usage opportunities [7]. Internet skills affect the types of activities performed online and play a crucial role in translating uses into actual outcomes [10]. All stages have their own grounds of determination, interact to shape cumulative digital inequalities, and directly affect tangible health outcomes [9,10]. We therefore hypothesize that:

- Hypothesis 1 (H1): Internet attitude is positively associated with (1) material access, (2) internet skills, (3) health-related internet use, and (4) health outcomes.
- H2: Material access is positively associated with (1) internet skills, (2) health-related internet use, and (3) health outcomes.
- H3: Internet skills are positively associated with (1) health-related internet use and (2) health outcomes.
- H4: Health-related internet use is positively associated with health outcomes.

### Health Statuses as Predictors of Internet Access

For the second goal of this paper, we focus on a range of health statuses pertaining to general functioning and well-being among the general population [11]. We first consider physical functioning, or the extent to which health interferes with a variety of functioning activities, such as participating in sports, carrying groceries, climbing stairs, or walking. Second, we consider social functioning, or the extent to which health interferes with normal social functioning activities, such as visiting friends. Third, mental health concerns one’s general mood, including depression, anxiety, and psychological well-being. Fourth, health perception involves one’s overall rating of current personal health. Finally, we consider the extent of bodily pain. We expect that these health statuses affect the different stages of internet access, as several studies have shown high rates of health-related internet use among those with medical conditions [12]. However, evidence on this relationship is inconclusive [12], as other studies have revealed that people who self-report being in good health are more likely to use the internet for health information [13] and that poor health inhibits particular stages of internet access [1,14]. For the different health statuses, causation could be argued in both directions, as compromised health might result in health-related internet use to become informed about specific conditions but might also restrict, for example, the use of certain devices or the development of internet skills. As the main purpose of this study is to assess the relationship between health statuses and internet access, we pose the following nondirectional hypotheses:

- H5: Physical functioning is associated with (1) internet attitude, (2) material access, (3) internet skills, and (4) health-related internet use.
- H6: Social functioning is associated with (1) internet attitude, (2) material access, (3) internet skills, and (4) health-related internet use.
- H7: Mental health is associated with (1) internet attitude, (2) material access, (3) internet skills, and (4) health-related internet use.
- H8: Health perceptions are associated with (1) internet attitude, (2) material access, (3) internet skills, and (4) health-related internet use.
- H9: Pain is associated with (1) internet attitude, (2) material access, (3) internet skills, and (4) health-related internet use.

### Research Model

**Figure 1:**
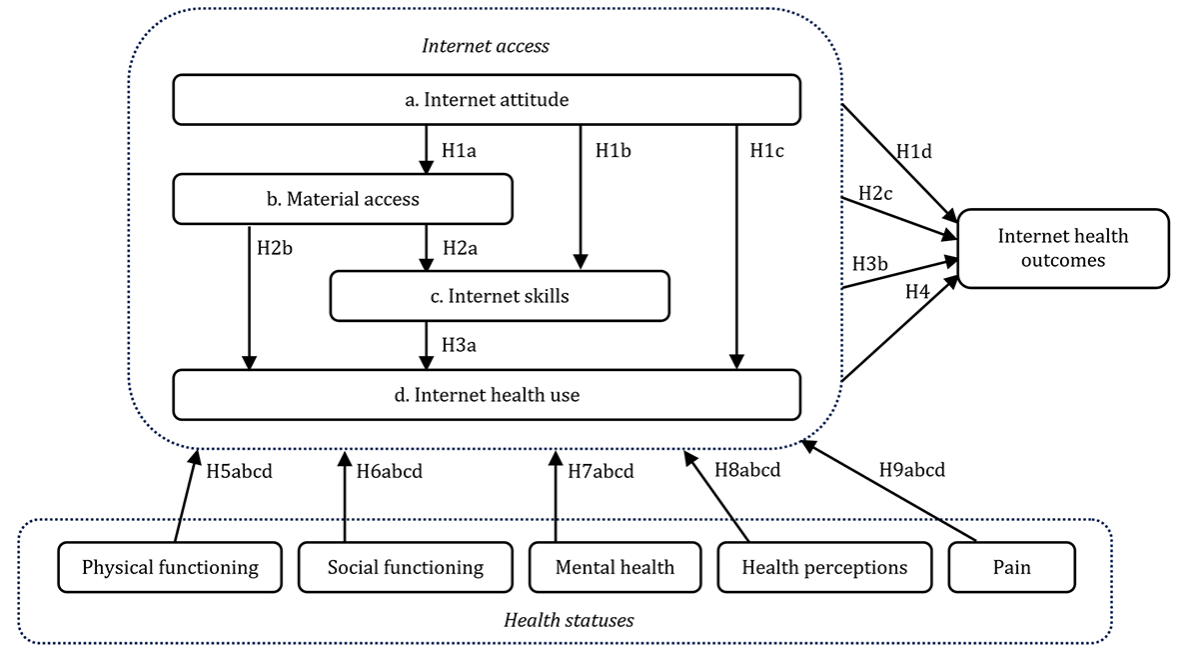
Conceptual model and hypotheses.

Figure 1 illustrates the research model built on the hypotheses. The model reflects resources and appropriation theory [6] by showing the core of the theory (the 4 phases of internet access) and considering personal categorical inequalities (in this contribution, the 5 health statuses). The internet health outcomes block reflects the potential benefits or outcomes from the 4 internet access phases.

### Moderating Effects of Age and Education

The conceptual model in Figure 1 shows that the different health statuses are expected to support or inhibit internet access and, as such, obtain positive health outcomes. In this study, we further focus on the moderating roles of age and education, which represent important and common types of segmentation in digital inequality research [5]. We study to what extent age and educational differences exist in the contributions of the 5 health statuses to internet access. We expect contributions to become stronger for users over 65 years of age (seniors) when compared with the overall population and for less-educated users when compared with those with higher levels of educational attainment. Elderly and less educated individuals are more likely to perceive and actually suffer from limited health status [15]. Further examination of the moderating effects of age and education on internet access is important to explain differences in internet health outcomes. This approach further supports the development of health information and services aimed at different age and educational groups and future planning of the health care system for these specific groups.

## Methods

### Recruitment

This study used online surveys and drew upon a sample collected in the Netherlands. To obtain a representative sample of the population, we used PanelClix, a professional organization for market research. Members of the panel receive a small incentive for every survey they complete. In the Netherlands, 98% of the population uses the internet, closely representing the general population in terms of sociodemographic composition. We aimed to obtain a data set with approximately 1700 respondents over the age of 18 years. Eventually, this resulted in the collection of 1730 responses in a 2-wave study, both conducted over a 1-week period. The survey in the first wave (April 2020; n=2227) was specifically designed to gather background variables, including the different health statuses that are the topic of interest in this contribution. The survey furthermore included questions related to COVID-19. The average time required to complete this survey was 15-20 minutes. The survey in the second wave (November 2020; n=1730, 77.7%) was administered among respondents of the first wave and involved questions around the different internet access stages, including internet motivation, material access, internet skills, internet health use, and health outcomes. The reason for administering a second survey among respondents of the first wave was a practical one: as the background variables were already collected, more space was available for questions related to internet access. Of the respondents of the first survey, 1730 (77.7%) completed the second survey. The average time required to complete this second survey was 20 minutes. During the first wave, 3 amendments to the sampling frame were made to ensure the representativeness of the Dutch population. Accordingly, the analyses revealed that respondents’ gender, age, and formal education largely matched official census data. This was also the case for the sample that resulted from the second wave. See Table 1 for an overview.

Both online surveys followed Mahon’s [16] recommendation to set an information sheet as the first page of the online survey in which potential respondents are required to check a box to indicate consent before accessing the survey. The survey used software that checked for missing responses and prompted users to respond. Both surveys were pilot-tested with 10 internet users over 2 rounds. Amendments were made based on the provided feedback. No major comments were provided in the second round.

**Table 1:** Characteristics of the study sample (N=1730).

Characteristics		Participants, n (%)
**Gender**		
	Male	871 (50.3)
	Female	859 (49.7)
**Age (years; mean 50.24, SD 17.02)**		
	18-34	397 (22.9)
	35-49	412 (23.8)
	50-64	502 (29.0)
	≥65	419 (24.2)
**Educational level**		
	No diploma, primary or lower secondary diploma	516 (29.8)
	Secondary diploma	602 (34.8)
	Higher diploma	612 (35.4)

### Ethical Considerations

To comply with requirements on privacy, collected data were anonymized by stripping IP addresses from the data set before the data files were saved to the researcher’s computer.

### Measures

*Internet attitude* was measured by 3 items adapted from the Digital Motivation Scale [17]. To measure *material access*, we considered a total of 7 different devices used to connect to the internet: desktop, laptop, tablet, smartphone, smart TV, game console, and smart device (eg, activity tracker; mean 3.43, SD 1.53). *Internet skills* were measured by the conceptual idea behind the Internet Skills Scale [9]. A 20-item measure was constructed in which items were scored on a 5-point scale. For health-related internet use, we used 6 items in which respondents were asked to indicate to what extent they used the internet for a particular online health activity. A 6-point scale was applied as an ordinal-level measure. Principal component analysis with varimax rotation was used to determine whether the items covered more underlying clusters, which was not the case. All items were retained in a single factor with an eigenvalue over 1.0, together accounting for 59% of the total variance. For *health outcomes*, we used 4 items that represent one’s satisfaction with health-related achievements. All constructs exhibited high internal consistency; see Table 2.

The measures for the 5 considered health statuses were adapted from the Dutch version of the Medical Outcomes Study (MOS) Short-Form General Health Survey (SF-20) [18]. This instrument enables respondents to assess their general health and generates composite summary scores representing different health status. With the exception of physical pain, we normalized the scales, with higher scores representing better functioning, for *physical functioning*, *social functioning*, *mental health*, *health perception*, and *physical pain* (Table 3).

Gender was included as a dichotomous variable, and age was directly asked. Data on education were collected by degree. These were subsequently divided into 2 groups of low (ie, no diploma or primary or [lower] secondary education diploma) and high (ie, college and university) educational level attained.

**Table 2:** Items, descriptive statistics, and internal consistency (Cronbach α) for internet attitude, internet skills, health-related internet use, and health-related internet outcomes.

Items		Mean (SD)
**Internet attitude (α=.74)^a^, mean 4.10, SD 0.70**		
	Technologies, such as the internet and mobile phones, make life easier.	4.29 (0.83)
	I feel that people pressure me to be constantly connected (recoded).	4.03 (1.23)
	There are many things on the internet that are good for people like me.	3.89 (0.85)
**Internet skills (α=.96)^b^, mean 3.45, SD 0.96**		
	I know how to upload files.	3.16 (1.07)
	I know how to adjust privacy settings.	3.54 (1.04)
	I know how to use my smartphone as a hotspot.	4.11 (1.55)
	I know how to check whether the information I find online is true.	3.33 (1.21)
	I find it easy to decide what the best keywords are.	4.17 (1.02)
	I know how to figure out whether a website can be trusted.	3.71 (1.22)
	I know how to store photos, documents, or other files in the cloud (eg, Google Drive, iCloud).	3.76 (1.41)
	I know how to keep track of the costs of mobile app use.	4.17 (1.41)
	I know how to change with whom I share content (eg, friends, friends of friends, or the public).	4.28 (1.13)
	I know how to block messages from people I do not want to have anything to do with anymore.	4.16 (1.14)
	I know what pictures of me or others I can share online.	4.23 (1.11)
	I know how to turn off my location on a smartphone.	3.24 (1.19)
	I know how to reach people with my digital creations.	3.66 (1.37)
	I know how to create videos or selfies to which others will react positively.	4.18 (1.32)
	I know how to create digital materials to express my ideas.	3.60 (1.35)
	I know how to block unwanted popup messages or ads.	3.59 (1.38)
	I know how to post homemade videos or music online.	3.64 (1.46)
	I know how to make basic changes to the content that others have produced.	3.48 (1.38)
	I know which (copy) rights apply to online material.	3.57 (1.29)
	I know how to increase the number of followers of my profile on social media.	3.45 (2.05)
**Health-related internet use (α=.86)^c^, mean 2.08, SD 0.86**		
	Finding information about your health or medical care	2.60 (1.02)
	Contacting a physician or medical specialist	1.94 (0.98)
	Talking to others about your personal health	1.91 (1.23)
	Participating in an online training or health program	1.76 (1.18)
	Finding information or watching videos about improving your fitness/health	2.05 (1.27)
	Using an app to check your health status or treatment	1.96 (1.33)
**Health-related internet outcomes (α=.85)^d^, mean 2.13, SD 1.39**		
	The way the last advice, program, or app you used affected your health	2.04 (1.56)
	The feeling about your fitness/health that online information gives you	2.23 (1.59)
	The latest online health information or online advice that you applied	3.03 (2.03)
	The way you have adapted your behavior based on online health information	2.11 (1.53)

^a^A 5-point agreement scale ranging from “strongly disagree” to “strongly agree.”

^b^A 5-point truth scale ranging from “not at all true of me” to “very true of me.”

^c^A 6-point frequency scale ranging from “never” to “multiple times a day.”

^d^A 5-point satisfaction scale ranging from “very dissatisfied” to “very satisfied.”

**Table 3:** Items, descriptive statistics, and internal consistency (Cronbach α) for health state variables.

Items		Mean (SD)
**Physical functioning (α=.89)^a^, mean 1.75, SD 0.34**		
	Vigorous activities, such as lifting heavy objects, running, or participating in strenuous sports	1.57 (0.50)
	Moderate activities, such as moving a table or carrying groceries	1.77 (0.42)
	Walking uphill or climbing a few steps without resting	1.74 (0.44)
	Bending or lifting or stooping	1.73 (0.44)
	Walking 1 block	1.83 (0.38)
	Eating, dressing, bathing, or using the toilet	1.89 (0.31)
**Social functioning^b^**		
	My health regularly limits me in social activities (eg, visiting friends or family)—recoded.	3.81 (1.16)
**Mental health (α=.85)^b^, mean 3.65, SD 0.77**		
	I regularly feel depressed and gloomy (recoded).	3.43 (1.05)
	I am often so sad that nothing can cheer me up (recoded).	3.60 (0.87)
	I am regularly nervous (recoded).	3.65 (1.10)
	I usually feel calm and composed.	3.66 (0.84)
	I feel happy most of the time.	4.05 (1.01)
**Health perception (α=.86)^b^, mean 3.39, SD 0.85**		
	I am a little sick (recoded).	3.72 (1.18)
	I am as healthy as anyone I know.	3.22 (1.04)
	My health is excellent.	3.28 (1.06)
	I have been feeling bad lately (recoded).	3.73 (1.05)
**Physical pain^c^**		
	Have you experienced any physical pain in the past 4 weeks?	3.67 (1.26)

^a^Did your health condition limit you in any of the following activities last year? If so, for how long? Yes, longer than 3 months/Yes, less than 3 months/No → transposed to No (1)/Yes (2).

^b^A 5-point agreement scale ranging from “strongly disagree” to “strongly agree.”

^c^A 5-point scale ranging from “heavy pain” to “no pain.”

### Statistical Analysis

To test the first hypothesized relationships, we applied path analysis with Amos 23 (IBM Corporation). To obtain a comprehensive model fit, we included the suggested indices by Hair et al [19]: the *χ*^2^ statistic, the ratio of *χ*^2^ to its *df*, the standardized root mean residual (SRMR<0.08), the Tucker-Lewis index (TLI>0.90), the comparative fit index (CFI>0.95), and the root mean square error of approximation (RMSEA<0.06). These fit indices are typically used to represent the 3 categories of model fit: absolute, parsimonious, and incremental. We added covariates between the health status variables. The correlations between internet attitude, material access, internet skills, internet health use, and health outcomes were not high enough to cause multicollinearity concerns. To test for moderator effects of age and education, we applied multigroup analyses. First, the model was estimated for each of the subgroups separately to confirm its acceptable fit for each group. Then, multigroup analysis was used to test the significance of the *χ*^2^ difference.

## Results

### Measurement Model and Hypotheses

**Figure 2:**
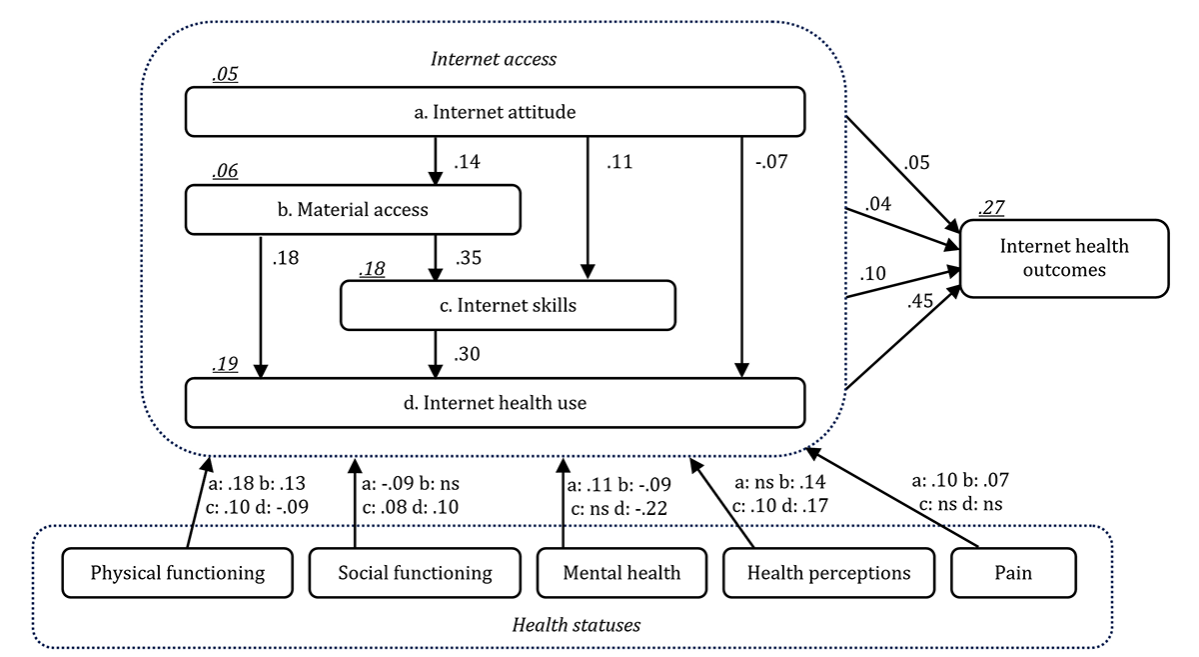
Structural model with path coefficients. Note: Path coefficients are significant at *P*<.05. Squared multiple correlations are underlined. ns: not significant.

To test the hypothesized relationships, we started by examining the basic assumptions of path analysis. Normality, kurtosis, and skewness did not differ significantly from acceptable criteria, and there were no outliers or multicollinearity beyond what would theoretically be expected. The structural model with coefficients and variances explained is presented in Figure 2. The results of the fit statistics indicated a good model fit: *χ*^2^_5_=23.68; *χ*^2^/*df*=4.74; SRMR=0.01; TLI=0.96; CFI=1.00, RMSEA=0.05 (90% CI 0.03-0.07). The magnitudes and significance of the direct, indirect, and total path coefficients are shown in Table 4. The significance of the indirect effects was examined using bootstrapping procedures [20] and the Monte Carlo method for assessing mediation [21,22].

The first hypotheses concerning the internet access stages and outcomes (H1-H4) are supported, with the exception of H1c. Internet attitude has a negative direct path to internet health use, and the total effect is 0. For the other hypotheses, all direct and indirect paths are positive and significant. See Table 4.

For the second set of hypotheses (concerning the health statuses), first Table 4 shows that physical functioning is directly and indirectly related to all 4 internet access stages (supporting H5a-d). Physical functioning contributes positively to internet attitude, material access, and internet skills but negatively to internet health use. Second, social functioning is directly and indirectly related to internet attitude, internet skills, and internet health use (supporting H6a,c,d). Social functioning contributes negatively to internet attitude and positively to internet skills and internet health use. There is a small indirect negative contribution to material access (partly supporting H6b). Third, the results revealed that mental health contributes positively to internet attitude and negatively to material access and internet health use (supporting H7a,b,d). There is no significant direct or indirect contribution to internet skills (rejecting H7c). Fourth, perceived health has a direct positive contribution to material access, internet skills, and internet health use (supporting H8b-d). There is no significant contribution to internet attitude (rejecting H8a). Finally, physical pain contributes positively to internet attitude and material access (supporting H8a,b). There are positive indirect contributions to internet skills and internet health use (partly supporting H8c,d).

**Table 4:** Significant direct, indirect, and total effects (standardizes regression weights and significance).

Path	Direct effects		Indirect effects		Total effects	
	β	*P* value	β	*P* value	β	*P* value
Internet attitude → health outcome	.05	.01	.03	.01	.08	.01
Material access → health outcome	.04	.04	.16	.01	.20	.01
Internet skills → health outcome	.10	.01	.13	.03	.23	.01
Internet health use → health outcome	.45	.02	N/A^a^	N/A	.45	.02
Internet attitude → material access	.14	.02	N/A	N/A	.14	.02
Internet attitude → digital skills	.11	.01	.05	.01	.16	.01
Internet attitude → internet health use	–.07	.03	.07	.01	.00	.50
Material access → internet skills	.35	.02	N/A	N/A	.35	.01
Material access → internet health use	.18	.01	.10	.01	.28	.01
Internet skills → internet health use	.30	.04	N/A	N/A	.30	.01
Physical functioning → internet attitude	.18	.02	N/A	N/A	.18	.02
Physical functioning → material access	.13	.01	.03	.01	.16	.02
Physical functioning → internet skills	.10	.01	.08	.01	.18	.01
Physical functioning → internet health use	–.09	.01	.07	.01	–.02	.62
Physical functioning → health outcomes	N/A	N/A	.03	.12	.03	.12
Social functioning → internet attitude	–.09	.01	N/A	N/A	–.09	.01
Social functioning → material access	.02	.49	–.01	.01	.01	.77
Social functioning → internet skills	.08	.03	–.01	.66	.07	.09
Social functioning → internet health use	.10	.01	.03	.07	.13	.01
Social functioning → health outcomes	N/A	N/A	.06	.02	.06	.02
Mental health → internet attitude	.11	.02	N/A	N/A	.11	.02
Mental health → material access	–.09	.02	.02	.01	–.07	.02
Mental health → internet skills	.01	.80	–.02	.26	–.01	.74
Mental health → internet health use	–.22	.01	–.02	.05	–.24	.01
Mental health → health outcomes	N/A	N/A	–.11	.02	–.11	.02
Perceived health → internet attitude	–.04	.36	N/A	N/A	–.04	.36
Perceived health → material access	.14	.02	–.01	.31	.13	.02
Perceived health → internet skills	.10	.03	.04	.01	.14	.02
Perceived health → internet health use	.17	.01	.07	.02	.24	.02
Perceived health → health outcomes	N/A	N/A	.12	.02	.12	.02
Physical pain → internet attitude	–.10	.01	N/A	N/A	.10	.01
Physical pain → material access	–.07	.01	–.01	.02	–.08	.01
Physical pain → internet skills	–.01	.65	–.04	.01	–.05	.09
Physical pain → internet health use	–.02	.65	–.03	.03	–.05	.19
Physical pain → health outcomes	N/A	N/A	–.04	.02	–.04	.02

^a^ N/A: not applicable.

### Moderator Effects

We tested for the significance of the *χ*^2^ difference between 2 specified age groups (<65 and ≥65 years) and between 2 educational groups (low and high). The results showed that for both age (*χ*^2^/*df*=3.716, *P*<.001, TLI=0.946, CFI=0.994, RMSEA=0.04 [95% CI 0.03-0.05]) and education (*χ*^2^/*df*=2.944, *P*<.001, TLI=0.962, CFI=0.996, RMSEA=0.03 [95% CI 0.02-0.05]), there are moderation effects on the overall model *χ*^2^. Table 5 shows the results of the direct path coefficient comparison between the 2 age groups and between the 2 educational groups.

Concerning age and internet access, Table 5 shows that the direct path coefficients from internet attitude to material access and internet skills are significantly larger for seniors. Furthermore, internet attitude contributes negatively to internet health use and positively to health outcomes in the group aged below 65 years. The contribution of material access to internet skills is slightly larger in the senior group, and the contribution of internet skills to internet health use is slightly smaller. In terms of age and the different health statuses, Table 5 reveals that physical functioning contributes positively to internet attitude, material access, and internet skills and negatively to internet health use in the group aged under 65 years. In the oldest age group, physical functioning contributes only positively to internet attitude. Social functioning contributes negatively to internet attitude and positively to internet skills and internet health use in the group aged under 65 years, while there are no direct significant contributions in senior group. In the group aged under 65 years, mental health contributes positively to internet attitude and negatively to material access and internet health use. In the senior group, there are positive contributions to material access and internet skills. The negative contribution of mental health to internet health use is significantly larger in the younger group. In this group, perceived health contributes negatively to internet attitude and positively to material access, internet skills, and internet health use. In the senior group, there is a negative contribution to material access. Finally, physical pain contributes negatively to internet attitude in both age groups, to material access in the younger group, and to internet health use in the senior group.

For education and internet access, Table 5 shows that the magnitude of the contribution of internet attitude to material access is larger among the less educated. Furthermore, internet attitude contributes positively to internet skills in this group. The contribution of material access to internet skills is also larger in the lower-educated group, while the contribution to internet health use is larger in the higher-educated group. The contribution of internet skills to internet health use is larger in the less-educated group. In relation to the different health statuses, the results showed that physical functioning contributes significantly more to internet attitude in the lower-educated group, while the positive contribution to material access is larger in the higher-educated group. For social functioning, there is a negative effect on internet attitude and a positive effect on material access in the higher-educated group. In the lower-educated group, there are positive effects on internet skills and internet heath use. Concerning mental health, the positive contribution to internet attitude and the negative contribution to material access are larger in the higher-educated group. For perceived health, in the higher-educated group, there is a significant effect on material access. Furthermore, there is a larger significant effect on internet health use in the higher-educated group. Finally, in the higher-educated group, there is a negative effect of physical pain on material access.

**Table 5:** Direct path coefficient comparisons for age and education.

Path	Age<65 years		Age≥65 years		Low education level		High education level	
	β	*P* Value	β	*P* Value	β	*P* Value	β	*P* Value
Internet attitude → health outcome	.06	.01	.03	.46	.03	.27	.08	.02
Material access → health outcome	.04	.18	–.00	.93	.05	.06	–.01	.74
Internet skills → health outcome	.04	.12	.11	.02	.07	.01	.12	<.001
Internet health use → health outcome	.46	<.001	.42	<.001	.45	<.001	.48	<.001
Internet attitude → material access	.13	<.001	.24	<.001	.21	<.001	.13	.002
Internet attitude → internet skills	.11	<.001	.14	.003	.14	<.001	.01	.77
Internet attitude → internet health use	–.08	.003	.08	.10	–.05	.06	–.07	.08
Material access → internet skills	.28	<.001	.31	<.001	.36	<.001	.29	<.001
Material access → internet health use	.16	<.001	.15	.002	.16	<.001	.21	<.001
Internet skills → internet health use	.26	<.001	.23	<.001	.30	<.001	.25	<.001
Physical functioning → internet attitude	.18	<.001	.19	.01	.21	<.001	.10	.04
Physical functioning → material access	.08	.03	.13	.08	.10	.01	.16	<.001
Physical functioning → internet skills	.08	.01	.02	.80	.10	.01	.10	.04
Physical functioning → internet health use	–.10	.003	–.11	.10	–.08	.04	–.11	.02
Social functioning → internet attitude	–.13	<.001	.03	.68	–.00	.94	–.25	<.001
Social functioning → internet access	.05	.21	–.08	.24	–.06	.15	.19	<.001
Social functioning → internet skills	.07	.04	.13	.07	.10	.01	.06	.31
Social functioning → internet health use	.11	.002	.01	.91	.10	.01	.08	.12
Mental health → internet attitude	.17	<.001	–.03	.64	.10	.01	.14	.003
Mental health → material access	–.10	.002	.14	.01	–.08	.02	–.12	.01
Mental health → internet skills	.01	.71	.12	.02	–.03	.33	.09	.06
Mental health → internet health use	–.21	<.001	–.16	.004	–.21	<.001	–.22	<.001
Perceived health → internet attitude	–.10	.03	.08	.31	–.04	.45	–.06	.34
Perceived health → material access	.19	<.001	–.11	.02	.08	.10	.24	<.001
Perceived health → internet skills	.12	.01	.08	.26	.11	.01	.09	.16
Perceived health → internet health use	.18	<.001	.10	.20	.15	<.001	.20	<.001
Physical pain → internet attitude	–.09	.01	–.12	.04	–.10	.01	–.11	.02
Physical pain → material access	–.09	.01	–.03	.59	–.04	.22	–.13	.01
Physical pain → internet skills	–.01	.71	–.00	.94	–.01	.78	–.02	.63
Physical pain → internet health use	–.00	.99	–.11	.05	–.01	.78	–.04	.44

## Discussion

### Principal Findings

This paper aimed to provide a comprehensive view of digital inequality in relation to different health statuses among the Dutch population. The study’s first goal was to reveal to what extent the process of internet access is important to obtain health outcomes. Internet attitude increases the likelihood of improving material access, the development of internet skills, and internet health use, suggesting that making online health apps attractive for larger segments of the population is an important objective. Material access, considered in this study as the diversity of the devices used, is highly relevant, as it has significant relationships with internet skills and internet health use. Individuals with different devices to connect to the internet everywhere and at all times of the day have more opportunities to develop internet skills and use online health apps. Internet skills are, in turn, required to use online health apps. The sequential nature of the access stages does not suggest that improving material access will automatically result in better internet skills or that a high level of internet skills will automatically result in a large variety of health-related internet use; all stages are, however, necessary conditions. The results furthermore revealed that all 4 access stages directly contribute to obtaining positive health outcomes, suggesting that to make online health care attainable for the general population, interventions should focus *simultaneously* on all stages. For example, attitudes might be improved by considering issues of accessibility and usability of online health information and services, material access by offering schemes such as device donation, internet skills by training programs tailored to the needs of people with different health statuses, and online health apps by awareness programs. Such approaches would require government, public, private, and nonprofit sector organizations to collaborate.

The second goal of this paper was to reveal to what extent different health statuses among the general population relate to the internet access stages and thus to internet health outcomes. The results confirmed that digital inequality research would benefit from considering health as a predictor of internet attitude, material access, internet skills, internet health use, and health outcomes. However, a general conclusion is that we should go beyond single self-reported measures of health, as different health statuses among the general population make unique contributions to the different internet access stages:

- Physical functioning contributes to internet attitude, material access, and internet skills, likely because physical limitations impact the process of taking up or learning how to use technologies (eg, in the case of smaller tablets or smartphones) [23]. Those with better physical functioning make less use of online health information and services as they have a relatively low need. Similarly, people with specific diseases that hinder physical functioning have less information need about their disease if they experience less limitations (eg, in the case of rheumatoid arthritis) [24].
- Better social functioning contributes to better material access and higher levels of internet skills. The importance of social bonds to use technology has long been established [25], and support from family, friends, or those that are important to the individual’s life contributes to learning to use a device or improving internet skills [26]. This is further strengthened when mobile phones, tablets, or laptops further enhance social connections and communication. Note that for internet skills, research has shown that informal support mainly works to apply basic skills [27]. The use of online health information and services is higher for those with poorer social functioning. This suggests that those whose health restricts people from visiting friends and family are more likely to seek health information online. This might be the result of a higher need for online health information and services but also of online health information serving as a substitute for information received from peers.
- Concerning mental health, the results revealed a positive contribution to internet attitude but a negative contribution to material access. An explanation might be that those suffering from mental health issues are more likely to experience excessive internet use, which is supported by the use of multiple devices to provide instant access at all times [28]. Furthermore, mental health negatively contributes to internet health use. As mental health is reflective of general distress, it causes people to turn to the internet for health information and services [29], apparently despite their less positive attitude toward the internet.
- People who perceive their health as higher have greater levels of material access and internet skills. A possible explanation might be that higher health perceptions foster social interactions that are supported by material access and higher levels of internet skills in the case of online social networking. The higher use of online health information and services among those with higher health perceptions seems to be inconsistent with prior research [30]. This discrepancy might be related to the influence of the COVID-19 pandemic in the survey period.
- Like poor physical functioning, physical pain negatively affects internet attitude and material access, suggesting that physical pain limits the use of certain devices and the process of learning how to use the internet.

In relation to our third goal, the general conclusion is that the contributions of the health statuses to the internet access stages differ for age and education. The main findings concerning age are that for seniors:

- internet attitude plays a more important role in obtaining material access than for those aged under 65 years. An important reason for seniors not to go online is a less favorable attitude toward the internet [31]. A positive, guided experience with the internet might motivate seniors to move to the following stages of internet access [31]. Furthermore, seniors are most likely to benefit most from accessible and usable apps [32].
- mental health plays a larger role in obtaining material access and developing internet skills. This suggests that seniors with mental health issues have a relatively high need for support, a worthwhile finding as online health interventions can reduce their mental health problems [33].
- perceived poor health hinders material access, suggesting that seniors who believe they are in poor health consider this as a barrier to interact with computer devices. This is a missed opportunity, as smartphones, tablets, or laptops might also be used as tools to enhance their perceived health [34].

The main findings concerning education are that for those with *lower* levels of education:

- internet attitude plays a larger role in obtaining material access, consistent with prior research that showed that education positively affects internet attitude [9]. Similar suggestions discussed for seniors apply, although specific approaches will be required.
- physical functioning is relatively important for developing a favorable internet attitude. This might be explained by the fact that lower-educated individuals are more likely to suffer from limitations in physical functioning [35], which could hinder the process of taking up and learning how to use the internet.
- social functioning plays a relatively important role in the development of internet skills and the use of online health information and services. Unfortunately, lower-educated individuals are less likely to perceive higher levels of support in relation to health [36], making organizing access to support an important objective.
- perceived health is relatively important for the development of internet skills. This suggests that lower-educated people who believe they are in poor health are more in need for skills training to make use of online health information and services as compared to their higher-educated counterparts.

### Limitations

A few limitations should be noted. The first is the study’s cross-sectional design, which did not allow confirmation of causal inferences about the association between health statuses and internet access. Furthermore, we focused on the general population, and the baseline status of the different health statuses varied slightly. Effects might have been stronger when targeting more people with serious conditions in relation to the 5 health statuses, although that was not the purpose of this study. Finally, we encourage further qualitative research to focus on the barriers and facilitators for people with different health statuses when using the internet to support their health needs.

### Conclusion

To obtain positive health outcomes and make online health care attainable for the general population, interventions should focus simultaneously on internet attitude, material access, internet skills, and internet health apps. However, issues of equality need to be considered and digital inequality research would benefit from considering health as a predictor of all 4 internet access stages and health outcomes. Furthermore, studies among the general population should go beyond single self-reported measures of health as physical functioning, social functioning, mental health, perceived health, and physical pain all demonstrated unique contributions to the internet access stages. The general conclusion is that different health statuses affect internet access stages in different ways and, consequently, the health-related opportunities that the internet offers. Further complicating this issue is that such influence is moderated by age and education.

## Abbreviations

CFI: comparative fit index
RMSEA: root mean square error of approximation
SRMR: standardized root mean residual
TLI: Tucker-Lewis index
